# Composition and Natural History of Snakes from Etá Farm region, Sete Barras, south-eastern Brazil

**DOI:** 10.3897/zookeys.931.46882

**Published:** 2020-04-30

**Authors:** Bruno F. Fiorillo, Bruno R. da Silva, Frederico Alcântara Menezes, Otavio A.V. Marques, Marcio Martins

**Affiliations:** 1 Programa de Pós-Graduação em Ecologia Aplicada, Escola Superior de Agricultura Luiz de Queiroz, Universidade de São Paulo. CEP 13418900, Piracicaba, SP, Brazil; 2 Laboratório de Coleções Zoológicas. Instituto Butantan, Av. Vital Brazil, 1500, São Paulo, SP, 05503-900, Brazil; 3 Departamento de Biologia Animal. Universidade Federal de Viçosa, Avenida P.H. Rolfs, s/n°, Campus Universitário, CEP 36571-000, Viçosa, Minas Gerais, Brazil; 4 Laboratório de Ecologia e Evolução. Instituto Butantan, Av. Vital Brazil, 1500, São Paulo, SP, 05503-900, Brazil; 5 Departamento de Ecologia. Instituto de Biociências, Universidade de São Paulo, 05508-090, São Paulo, São Paulo, Brazil

**Keywords:** banana plantation, diet, habitat, peach palm plantation, rainforest, reproduction, reptiles

## Abstract

Approximately 140 snake species are known to occur in the Atlantic Forest with nearly half being endemic to this ecoregion. However, the Atlantic forest is one of the most threatened tropical ecoregions, with only 16% of its original area remaining as forest. This extensive habitat loss must have had a negative effect on its snake fauna. Indeed, 53% of the threatened snakes of Brazil occur in the Atlantic forest. Therefore, basic natural history information that can potentially contribute to the conservation of Atlantic forest snakes are urgently needed. Here the natural history of a snake assemblage at Etá Farm region, Sete Barras municipality, south-eastern Brazil is described, and a visual guide and an identification key provided that can be used by researchers and local people to identify snakes from this region. Most of the species found in the field use both open areas and forests, are primarily terrestrial, present diurnal activity, and include frogs in their diet. A higher number of enlarged follicles, eggs, and/or embryos were recorded during the warm and rainy season. Seventeen different types of defensive tactics were recorded in the species found in the field. This study provides useful information for understanding the structure of snake assemblages of the Atlantic Forest and is potentially useful for conservation assessments and for designing conservation plans.

## Introduction

Natural history information, what organisms do in their respective environments, including interactions between them (Greene 1994), contributes beyond the basic refinement of science (Greene and McDiarmid 2005) but also to our understanding of how environments function and, consequently, aids in many aspects of conservation, management, and appreciation of nature (Caughley 1994, Brooks and McLennan 2002, Dayton 2003). Despite their obvious relevance, there is still a considerable knowledge gap on the ecology and behaviour of most snake species, even in well-studied regions in the world (Greene 2005). For example, *Bothrops jararacussu* is one of the most widespread species in the Atlantic forest, yet most of its natural history data come from only a few localities of southeastern Brazil (Marques 1998, Martins et al. 2002, Hartmann et al. 2009b). Valuable natural history information is available for only a small fraction of animal species, usually those that are large or common and relatively easy to study (Greene 1994). Neotropical snakes are no exception and despite the studies published on these animals (Strüssmann and Sazima 1993, Marques 1998, Martins and Oliveira 1998, Cechin 1999, Di-Bernardo 1999, Sawaya et al. 2008, Hartmann et al. 2009ab, Pontes et al. 2009, Gaiarsa et al. 2013, Mesquita et al. 2013, Guedes et al. 2014), many species are still only known from small portions of their distributions.

The Atlantic forest of eastern Brazil harbours a very rich snake fauna, with approximately 140 species, representing 34% of the 412 species of snakes known to occur in Brazil (Costa and Bérnils 2018; Marques et al. 2019; Nogueira et al. 2019). Furthermore, almost half (45%) of the Atlantic forest snakes are endemic to these forests. However, the Atlantic forest is one of the most threatened tropical ecoregions (Myers et al. 2000), with only 16% of its original area remaining as forest (Ribeiro et al. 2009). The extensive habitat loss to which the Atlantic forest was subject in the last decades have likely had a negative effect on its snake fauna. Indeed, 53% of the threatened snakes of Brazil occur in the Atlantic forest and there is a lack of baseline data for an additional ten snake species, from this ecoregion, making the assessment of their conservation status difficult (ICMBio, 2018). Therefore, basic natural history information that can potentially contribute to the conservation of Atlantic forest snakes are urgently needed.

Here we provide basic natural history information for an Atlantic Forest snake assemblage from south-eastern Brazil. We sampled pristine along with disturbed habitats, thus assessing the ability of the Atlantic forest snakes to persist in disturbed habitats. For each species we provide primary information on habitat and micro-habitat use, time of activity, feeding habits, reproduction and defence. We also provide a short review of the natural history of each species based on our results and on previously published accounts.

## Materials and methods

**Figure 1.**
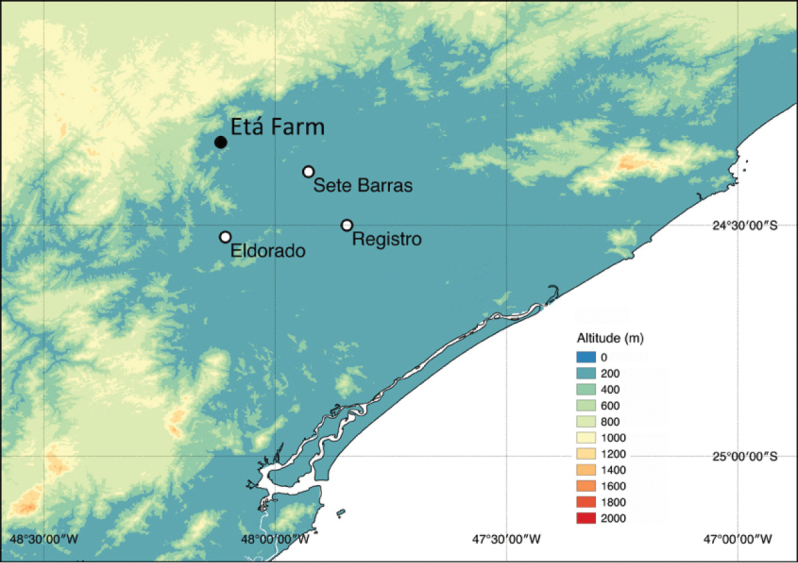
Topographic map of the region where the Etá Farm is located.

The primary information used in this study was obtained between April 2013 and March 2014 at the region of Etá Farm (24°19'13"S, 48°7'3"W) in the Sete Barras municipality, São Paulo state, south-eastern Brazil. The area is located within the Atlantic forest in a hillside forest formation (Joly et al. 1992). While this region shows great variation in elevation, ranging from 45 m at Etá Farm to over 800 m at the Sete Barras Operational Centre of Carlos Botelho State Park (Forlani et al. 2010), sampling for this study was carried out within the 45–80 m range (Figure 1). We searched the literature and museum databases for additional species that might occur in the Sete Barras municipality and neighbouring areas and that we could have failed to find during our fieldwork.

**Figure 2.**
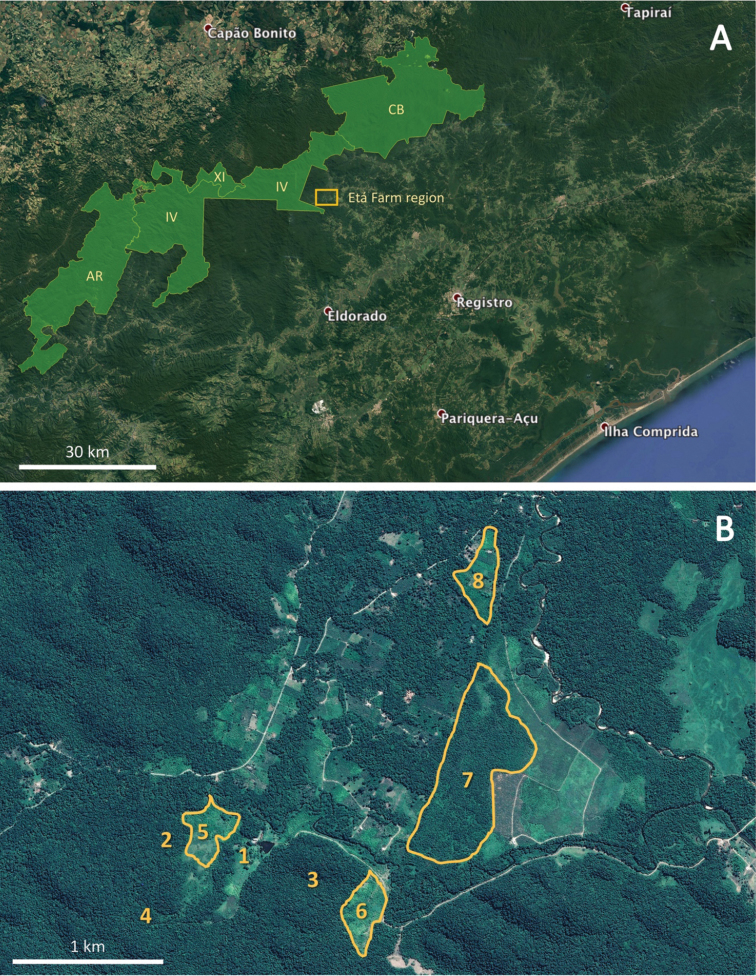
Satellite images (source: Google Earth) **A** the region where the study area (light orange rectangle) is located in the southern São Paulo State and the group of continuous protected areas (in light green) that encompasses most of the primary forests of this region (AR = Parque Estadual Turístico do Alto Ribeira; CB = Parque Estadual Carlos Botelho; IV = Parque Estadual Intervales; XI = Estação Ecológica de Xituê) as well as the location of the Etá Farm region (rectangle) **B** the region of the Etá Farm, Sete Barras Municipality where 1 indicates the Etá Farm administrative buildings, 2–4 indicate areas of forest, 5 and 6 indicate peach palm plantations; 7 indicates abandoned banana plantation, and 8 indicates an agricultural settlement.

Phytosociological and floristic studies have shown high richness of tree and shrub species in this type of Atlantic forest formation (Mantovani 1993). In addition to forests in an advanced stage of regeneration, areas modified by agriculture (considered “open areas”), such as peach palm (*Bactris gasipaes*) and banana (*Musa* sp.) plantations, were also sampled (Figure 2).

Field data were collected by two researchers for 14 days per month, from April 2013 to March 2014, for a total sample time of 168 days. Snakes were sampled with pitfall traps with drift fences (Greenberg et al. 1994, Cechin and Martins 2000), time constrained searches (sensu Campbell and Christman 1982, Scott et al. 1989, Martins and Oliveira 1998), and accidental encounters (Martins and Oliveira 1998), the latter including snake encounters by local people (Martins and Nogueira 2012). Three main vegetation types were sampled with pitfall traps with drift fence: banana plantation (Figure 2B; site 7), peach palm plantation (Figure 2B; sites 5 and 6), and forest (Figure 2B; sites 2 and 3). Our sampling design for pitfall traps included two sampling units per vegetation type, each sampling unit comprising three Y sets (with 12 m-long branches), located 100 m from each other. Thus, we installed a total of six sampling units with a total of 18 Y sets and 72 buckets. Sampling units were located at least 500 m from each other. Each Y set had four 100 L plastic buckets (three at each branch end and one in the centre) connected by a 60 cm-high plastic fence. The buckets were perforated at the bottom to avoid accumulation of rainwater. Two additional sites (Figure 2B; sites 4 and 8) were sampled occasionally or through time constrained searches.

Habitat use was recorded through active search for individuals (made only at night), describing the habitats (e. g., open area, forest, banana plantation) and micro-habitats used by each snake (fossorial, aquatic, terrestrial or arboreal) and perch height (in case of arboreal species). To characterise micro-habitats we used only information obtained during active searches; for individuals collected with pitfall traps, only vegetation cover (forest, peach palm or banana plantation) was considered.

To describe diet, collected specimens were dissected through an incision in the ventral region. Food items were identified to the lowest possible taxonomic rank using taxonomic keys, identification guides, specimens deposited in scientific collections and help from experts. Whenever the prey came from a snake captured in a pitfall trap, this information was included, given the possibility of the snake having ingested prey that had also fallen in the trap (Cechin and Martins 2000) but which is not part of the snake’s usual diet. Additional specimens of the studied species from the herpetological collection of the Butantan Institute were also dissected.

To describe reproductive condition, we recorded the length of the largest follicle, egg or embryo, and number of vitellogenic follicles (> 10 mm), eggs or embryos in every month of collection. Specimens collected in the field in the study area and specimens from the herpetological collection of the Butantan Institute were dissected for this purpose.

Behavioural descriptions are based on observations made over short periods of time (*ad libitum* and sequence samplings; Altmann 1974). Defensive behaviours were recorded when individuals were observed in the field and when handled.

## Results

With a sampling effort of 168 days of fieldwork, including 558 person-hours of visual search, we found 255 individuals of 17 species of snakes (14 genera, four families) at the Etá Farm region. Additionally, we included *Corallus cropanii* to our study because it was found previously by other researchers in our study area (Machado Filho et al. 2011). Species richness was similar between forests (13 species) and disturbed areas (banana plantation, peach palm plantation, roads, pastures, and around houses; 16 species; Table 1).

**Table 1.** Number of individual snakes found in the Etá Farm region, Sete Barras, SP, Brazil, in forests and disturbed areas, considering all sampling methods. Forest includes forests and forest borders; Disturbed includes banana plantations, peach palm plantations, and other disturbed areas (roads, pastures, areas around houses); *N* = number of specimens recorded. The asterisk indicates a species that was found by other researchers in our study area (Machado-Filho et al. 2011).

	Forest	Disturbed	*N*
** Boidae **			
Corallus cropanii*		1	1
** Colubridae **			
*Chironius exoletus*		1	1
*Chironius fuscus*	1	7	8
*Chironius laevicollis*	3	1	4
*Spilotes pullatus*		15	15
** Dipsadidae **			
*Dipsas neuwiedii*		25	25
*Echinanthera cephalostriata*	2	1	3
*Erythrolamprus aesculapii*	2	3	5
*Erythrolamprus miliaris*	22	53	75
*Helicops carinicaudus*	7	6	13
*Oxyrhopus clathratus*	3	9	12
*Sordellina punctata*	5	2	7
*Taeniophallus bilineatus*	2		2
*Tomodon dorsatus*		3	3
*Xenodon neuwiedii*	2	4	6
** Elapidae **			
*Micrurus corallinus*	1	5	6
** Viperidae **			
*Bothrops jararaca*	4	19	23
*Bothrops jararacussu*	9	38	47
**TOTAL**	**63**	**193**	**256**

**Figure 3.**
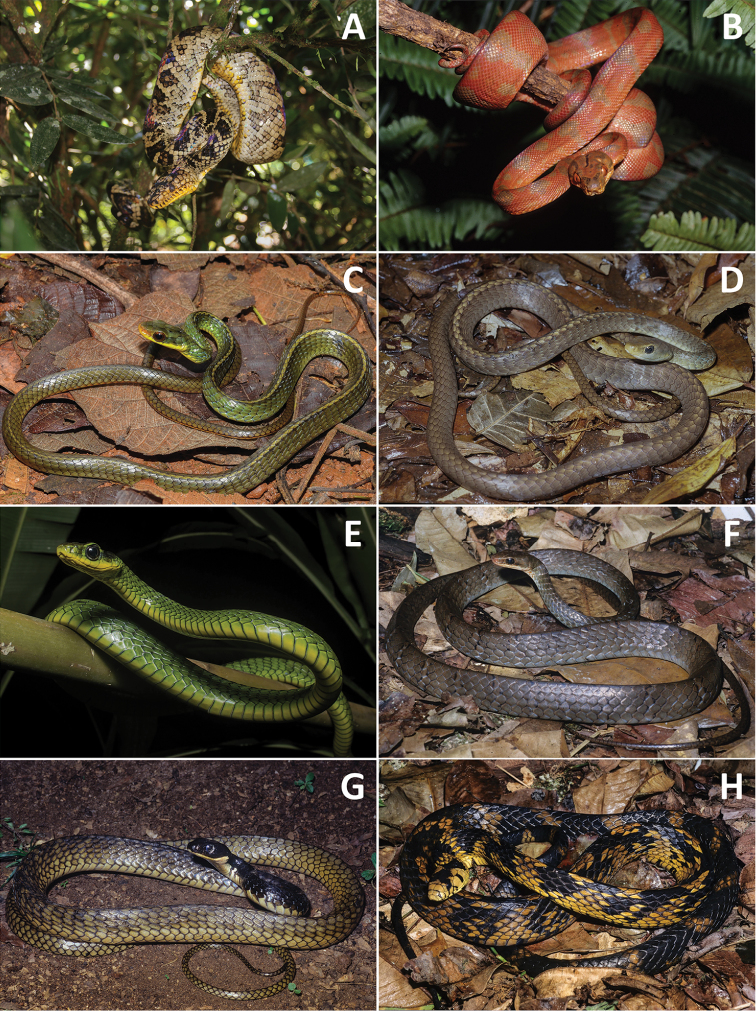
**A***Corallus cropanii***B***C. hortulanus***C***Chironius bicarinatus* (photo: Arthur Abegg) **D***C. exoletus***E***C. foveatus***F***C. fuscus***G***C. laevicollis***H***Spilotes pullatus*.

**Figure 4.**
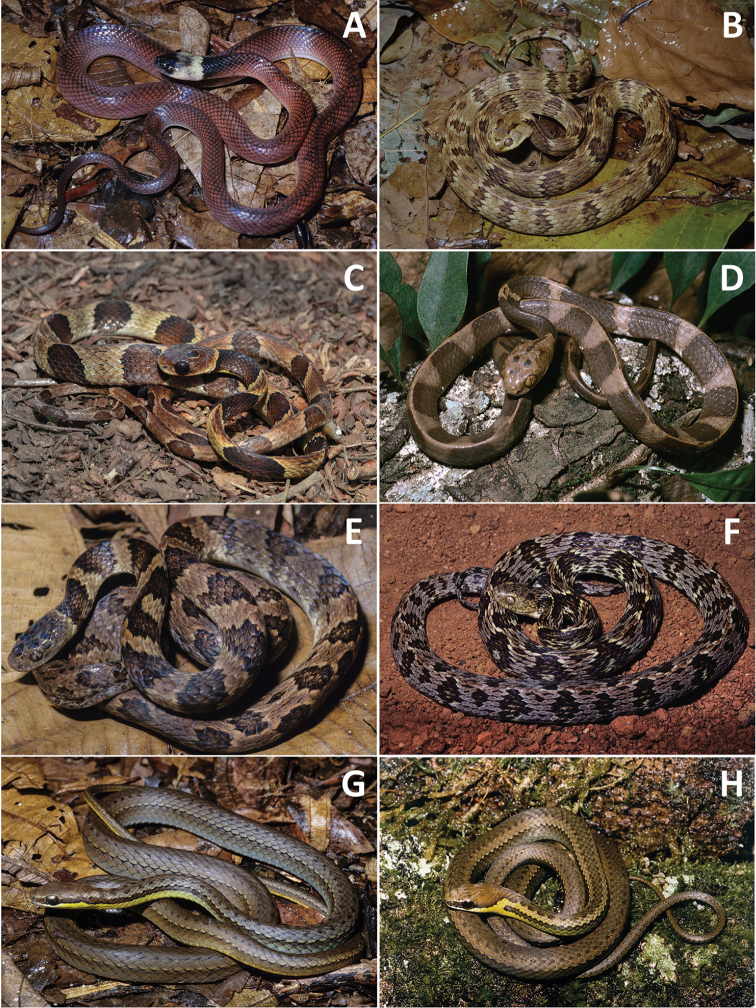
**A***Clelia plumbea***B***Dipsas albifrons***C***D. alternans* (photo: Arthur Abegg) **D***D. indica petersi***E***D. neuwiedi***F***D. variegata***G***Echinanthera cephalostriata***H***E. cyanopleura* (photograph Marcos Di-Bernardo).

**Figure 5.**
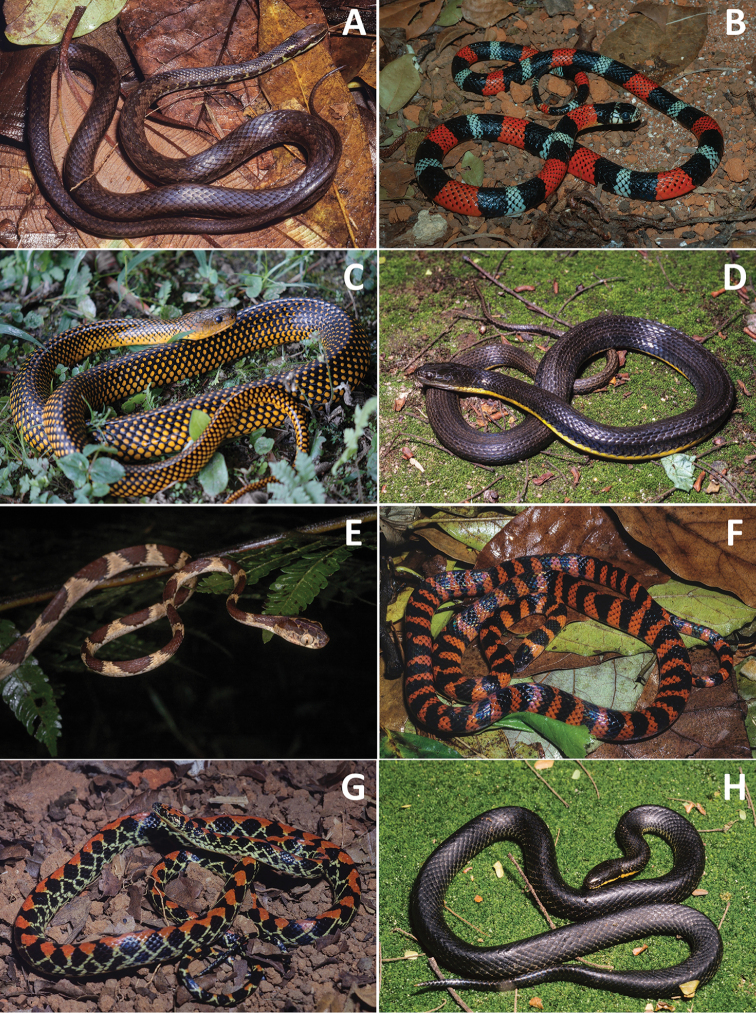
**A***Echinanthera undulata***B***Erythrolapmprus aesculapii***C***E. miliaris***D***Helicops carinicaudus***E***Imantodes cenchoa* (photo: Ricardo J. Sawaya) **F***Oxyrhopus clathratus***G***Siphlophis pulcher***H***Sordellina punctata*.

**Figure 6.**
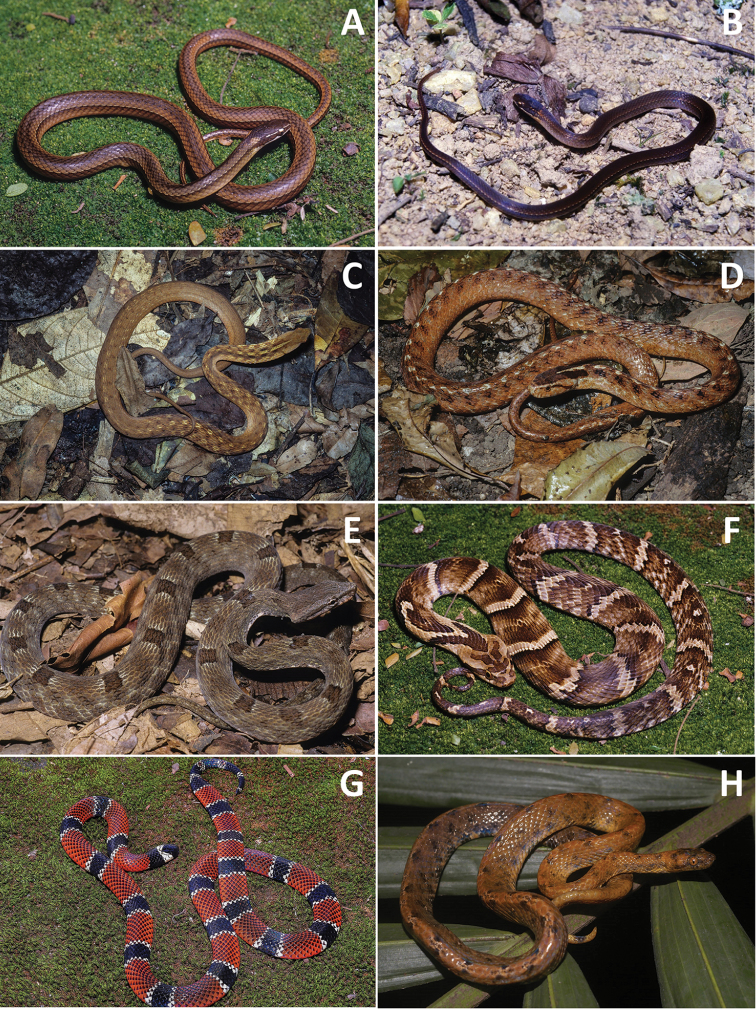
**A***Taeniophallus bilineatus***B***T. persimilis***C***Thamnodynastes nattereri***D***Tomodon dorsatus***E***Tropidodryas serra***F***Xenodon neuwiedii***G***Micrurus corallinus***H***Tropidophis paucisquamis*.

**Figure 7.**
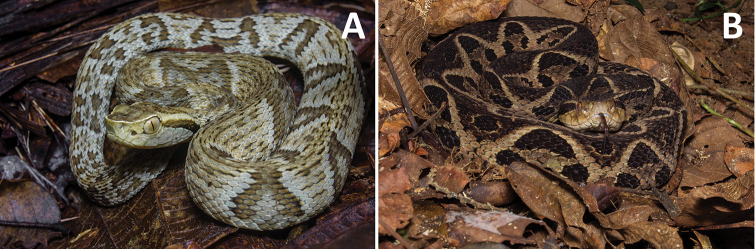
**A***Bothrops jararaca* (photo: Rafael Menegucci) **B***B. jararacussu*.

Besides the 17 species we found during our fieldwork at Fazenda Etá region and *C. cropanii* (Machado Filho et al. 2011), eight additional species are known to occur in the Sete Barras municipality: *Chironius bicarinatus*, *Chironius foveatus*, *Clelia plumbea*, *Dipsas albifrons*, *D. alternans*, *Echinantera undulata*, *Tropidodryas serra*, and *Tropidophis paucisquamis* (Nogueira et al., 2019). Furthermore, eight additional species occur in neighbouring regions (Cananéia Island, Iguape, Registro, and Pariquera-Açu) and thus could also occur in the Fazenda Etá region: *Corallus hortulanus*, *Dipsas indica*, *D. variegata*, *Echinanthera cynopleura*, *Imantodes cenchoa*, *Siphlophis pulcher*, *Taeniophallus persimilis*, *and Thamnodynastes nattereri* (Sena 2007; Pereira et al., 2007; Nogueira et al, 2019). We included all the species above in the figures depicting the snakes found in the Etá Farm region (Figs 3–7), as well as in the identification key here provided, because they can be used by researchers and local people to identify snakes they find in this region.

### General natural history patterns

Among the species we found in the field, most used forested areas (> 70% of species), were primarily terrestrial (70%), showed diurnal activity (> 58%), and included frogs in their diet (> 50%; information supplemented with data from the literature). Only those which consumed endothermic prey and *Dipsas neuwiedi* showed nocturnal activity. However, there was a relatively high percentage (30%) of semi-arboreal species, observed almost exclusively in open areas or forest edges, all anuran specialists (except for *Spilotes pullatus*) and belonging to the family Colubridae.

In addition to species that were semi-arboreal and anuran specialists, the mollusc-specialist species *D. neuwiedi* and *T. dorsatus* were also found exclusively in open areas. Only *T. bilineatus* proved to be the most limited to the forest habitat (*N* = 2), particularly to a cryptozoic micro-habitat (see Habitat Use of *Taeniophallus bilineatus* under Natural history accounts). The species *E. miliaris* and *B. jararacussu* showed the broadest spectrum of the assemblage in terms of resource use, as both widely used open and forested areas and included 3 and 4 different types of prey in their diet, respectively.

The viperids *B. jararaca* and *B. jararacussu* and the dipsadids *D. neuwiedii* and *O. clathratus* were the species most commonly found in disturbed areas such as plantations, around houses and even inside houses. Particularly for *O. clathratus*, 75% of observations occurred in disturbed habitats; the remaining took place in the forest.

Among the snake specimens whose stomach contents were analysed, 82 individuals, belonging to eleven different species, had prey in their digestive tracts. Of those, most have ingested frogs (61%), followed by mammals (18.3%), molluscs (11%), snakes (3.7%), fishes (2.4%), non-mollusc invertebrates (leech and centipede) (2.4%), and lizards (1.2%) (Table 2). The assemblage showed a great number of species whose bulk of the diet consisted of, or included, frogs, especially leptodactylids (more than 70% of anuran records), the group that was most captured in pitfall traps in the region (see Fiorillo et al. 2018). The second item most found was small mammals (more than 18%), of which rodents (more than 80% of the mammals), particularly from the tribe Akodontini (> 40% of the rodents) were the most frequent. Almost all small mammals recorded were consumed by vipers; only two cases were reported for species from other families (*S. pullatus* and *O. clathratus*).

**Table 2.** Food items found in the digestive tract of snakes from the region of Etá Farm region, Sete Barras, SP, Brazil. *N* = number of snakes with respective stomach or intestinal contents, or observation.

Family/Species	*N*	Stomach contents
** Colubridae **		
*Chironius laevicollis*	2^1^	*Leptodactylus latrans* (Anura, Leptodactylidae)^1^
*Spilotes pullatus*	1^1^	Unidentified rodent hair
** Dipsadidae **		
*Dipsas neuwiedi*	2^1^	Limax cf. flavus (Molusca, Limacidae)^1^
	1^1^	*Meghimatium pictum* (Molusca, Philomycidae)^1^
	2^1^	Unidentified Molusca^1^
	4^1^	*Phyllocaulis* sp. (Molusca, Philomycidae)^1^
*Erythrolamprus aesculapii*	1^1^	Snake scales
	1^1^	*Sibynomorphus neuwiedi* (Serpentes, Dipsadidae)^1^
*Erythrolamprus miliaris*	1^1^;1^2^	Unidentified frog fragments^2^
	1^1^;5^2^	Fragments of *Leptodactylus* sp. (Anura, Leptodactylidae)^1,2^
	2^2^	Fragments of *Rhinella* sp. (Anura, Leptodactylidae)^2^
	3^2^	*Leptodactylus latrans* (Anura, Leptodactylidae)^2^
	1^1^;4^2^	*Leptodactylus notoaktites* (Anura, Leptodactylidae)^1,2^
	1^1^; 14^2^	*Physalaemus spiniger* (Anura, Leptodactylidae)^1, 2^
	1^2^	*Placosoma glabellum* (Lacertilia, Gymnophtalmidae)^2^
	2^2^	*Rhinella hoogmoedi* (Anura, Leptodactylidae)^2^
	2^2^	*Rhinella icterica* (Anura, Bufonidae)^2^
	1^2^	*Rhinella ornata* (Anura, Bufonidae)^2^
	1^1^	*Synbranchus marmoratus* (Synbranchiformes, Synbranchidae)^1^
*Helicops carinicaudus*	1^1^	*Characidium* sp. (Characiformes, Crenuchidae)
	1^1^	Unidentified frog fragments
	1^1^	*Leptodactylus latrans* (Anura, Leptodactylidae)^1^
*Oxyrhopus clathratus*	1^2^	*Monodelphis americana* (Didelphimorphia, Didelphidae)^2^
*Sordellina punctata*	1^1^	Leech (Annelida, Hirudinea)^1^
*Xenodon neuwiedii*	1^1^	Unidentified frog fragments
	1^1^	Fragments of *Rhinella hoogmoedi* (Anura, Bufonidae)^1^
	1^2^	*Rhinella icterica* (Anura, Bufonidae) ^2^
** Viperidae **		
*Bothrops jararaca*	1^1^	Akodontini (Rodentia)^1^
	2^1^	Unidentified rodent hair
*Bothrops jararacussu*	1^1^	*Akodon* sp. (Rodentia, Cricetidae)^1^
	2^1^	Akodontini (Rodentia, Cricetidae)^1^
	1^1^	*Brucepattersonius* sp. (Rodentia, Cricetidae)^1^
	1^1^	Didelphis cf. aurita (Marsupialia, Didelphidae)^1^
	1^1^	Fragments of *Leptodactylus* sp. (Anura, Leptodactylidae)^1^
	3^1^	Fragments of *Leptodactylus latrans* (Anura, Leptodactylidae)^1^
	1^1^	Fragments of Hylidae (Anura, Leptodactylidae)^1^
	1^1^	Unidentified mammal fragments
	1^1^	Centipede (Scolopendromorpha, Scolopendridae)^1^
	1^1^	*Oligoryzomys* sp. (Rodentia, Cricetidae)^1^
	3^1^	Unidentified rodent
	1^1^	*Sordellina punctata* (SerpentesDipsadidae)^1^

^1^Individuals captured in active searches or by others. ^2^ Individuals captured in pitfall traps.

Although, in qualitative terms, *E. miliaris* and *B. jararacussu* showed a greater diversity of items in their diets, they may be considered specialists in frogs (> 95% of the diet of *E. miliaris*) and small mammals (> 58% of the diet of *B. jararacussu*), respectively. However, most of the records obtained for *E. miliaris* came from individuals caught in pitfall traps (> 87% of cases). Hence, part of the frogs may have been opportunistically consumed by this species (the finding of 14 specimens of *E. miliaris* captured in pitfall traps, that ingested *P. spiniger*, supports this assumption). Only four individuals of this species were captured by other capture methods, one of which had consumed a fish.

Of the specimens examined, 28 were reproductive females containing vitellogenic follicles, eggs or embryos. In the three families, as with activity, larger follicles were found during the Austral Spring, with the largest vitellogenic follicles, as well as eggs and embryos, occurring from September to October, except for one specimen of *Erythrolamprus aesculapii* that had vitellogenic follicles during the month of July (Austral Winter). Over the sampling period, only one single mating behaviour was observed for *S. pullatus*, in September (see Natural history accounts).

A total of 17 different defensive tactics was recorded for the assemblage studied, with some variations and combinations of them (Table 3). Most seem to be aimed at visually oriented predators (being “flatten body” most frequent among them, used by 70% of the species), but cloacal discharge had the same frequency (used by 70% of the species in the region, considering field observations and data from the literature). Another defence shown by many species was cryptic colouration (82%), which was common in diurnal species (58% of the assemblage). In contrast, only three species showed aposematic or mimetic colouration (*M. corallinus* and their supposed mimics *E. aesculapii* and *O. clathratus*).

**Table 3.** Defensive tactics of snakes from the Etá Farm region, Sete Barras, SP, Brazil. CB = compress body while raising head; CD = cloacal discharge; SC = S-coil; DM = display buccal mucosa; DV = display ventral posterior region; EM = perform erratic movements; FB = flatten body; HH = hide head; IG = inflate gular region; MI = mimicry; OM = open mouth; RB = rotate body; RH = raise head; ST = strike; TD = tail display; TH = triangulate head; VT = vibrate tail. The numbers indicate field observations and an “X” indicates data from the literature (Hoge 1953, Marques and Sazima 2004, Martins et al. 2008, Menezes et al. 2015).

Species	CB	CD	SC	DM	DV	EM	FB	HH	IG	MI	OM	RB	RH	ST	TD	TH	VT
*Chironius exoletus*		X	1						X		1			1			
*Chironius fuscus*		X	X						X		X	X	X	2			X
*Chironius laevicollis*		X	X						X		X		X	X			X
*Spilotes pullatus*		X	X						10		1		X	4			4
*Dipsas neuwiedi*		4	X					1		X			X			19	
*Echinanthera cephalostriata*		X					X						1				
*Erythrolamprus aesculapii*	X					X	X	X		X					X		
*Erythrolamprus miliaris*	X	20					2					5				3	
*Helicops carinicaudus*		X					X	X						4			
*Oxyrhopus clathratus*						X	1	X		X							
*Sordellina punctata*		2					X										
*Taeniophallus bilineatus*		X					X										
*Tomodon dorsatus*			X	X			X	X					X	X			
*Xenodon neuwiedii*	X		X				1			X			X	1		X	
*Micrurus corallinus*	X				1	X	X	X									
*Bothrops jararaca*		X					X							4			6
*Bothrops jararacussu*		X					X							2			5

### Natural history accounts

#### Boidae Gray, 1825

##### *Corallus cropanii* (Hoge, 1953)

This large species (maximum SVL = 1510 mm; Marques et al. 2019) is rare in the Etá Farm region. Although not sampled during our study, an individual of *C. cropanii* was found by Machado Filho et al. (2011) in the agricultural settlement north of the Etá Farm (Fig. 2). The holotype was found on vegetation at 1.5 m above the ground; in captivity, it remained perched on branches for most of the time (Marques and Cavalheiro 1998). The only known food content is the land opossum, *Metachirus nudicaudatus* (Didelphimorphia, Didelphidae; Marques and Cavalheiro 1998). This is a viviparous species (Marques et al. 2019), but there is no detailed information available on its reproduction. It may raise its head while opening its mouth (Hoge 1953).

#### Colubridae (Ooppel, 1811)

##### *Chironius exoletus* (Linnaeus, 1758)

This is an aglyphous species of medium size (maximum SVL = 790 mm; *N* = 1). Only one individual was found, in the peach palm plantation, on the vegetation at 0.5 m above the ground, during the day, in November. The available information indicates that it is semi-arboreal and diurnal (França and Araújo 2006, Martins et al. 2008, this study), but forage mainly on the forest ground where it feeds primarily on hylid frogs (Sazima 1992, Dixon et al. 1993, França and Araújo 2006, Rodrigues 2008; Marques and Sazima 2004; 2019). Its fecundity ranges from 4–12 eggs (Dixon et al. 1993, Bernarde and Abe 2006). When handled, the individual found opened its mouth, struck, and raised its head and formed an S-coil with the anterior part of the body (Table 3). It may also perform gular inflation, lateral fattening and cloacal discharge (Martins et al. 2008, Marques et al. 2019).

##### *Chironius fuscus* Linnaeus, 1758

This is an aglyphous species of medium size (maximum SVL = 919 mm; *N* = 8). It was found crossing an unpaved road, always near the forest edge (*N* = 6), and on a trail in the forest near the abandoned banana plantation, lying coiled up on a tree at 1.5 m above the ground (*N* = 1). One juvenile was found while crossing a paved road close to the urban area of Sete Barras, during the day. The available information indicates that it is diurnal and semi-arboreal, but forage mainly on the ground of the forests (Martins and Oliveira 1998, Marques and Sazima 2004, Martins et al. 2008, this study) where it feeds primarily on leptodactylid frogs (Strüssmann and Sazima 1993, Martins and Oliveira 1998, Marques and Sazima 2004). At Etá farm, it was found during most of the year, with a higher incidence of juveniles from April to June. One female had six vitellogenic follicles in May. In the Amazon region, females were collected with 3–8 eggs in the oviducts, from March–July and in October (Dixon and Soini 1986, Dixon et al. 1993, Martins and Oliveira 1998). The handled individuals struck (*N* = 2; Table 3). This snake also opens its mouth, raises its head, and forms an S-coil with the anterior part of its body, flattening and inflating the gular region in frontal display, performs cloacal discharge, rotates its body and vibrates its tail (Beebe 1946, Dixon and Soini 1986, Martins et al. 2008, Marques and Sazima 2004, Marques et al. 2019).

##### *Chironius laevicollis* (Wied, 1824)

This is an aglyphous species of large size (maximum SVL = 1650 mm; *N* = 4). One individual was found on the ground, during the day, moving through the peach palm plantation; another was foraging on the forest floor at the margins of the Etá River during the day. Before being captured, the individual found on the trail quickly climbed a tree to a height of approximately 3 m. One individual was observed in a shallow pond at the edge of the forest, “yawning” as if it had just ingested something, shortly before swimming away, also during the day. The available information indicates that it is diurnal and terrestrial, being arboreal during the juvenile stage (Dixon et al. 1993, Marques and Sazima 2003, Martins et al. 2008, this study). In the digestive tracts of the examined specimens we found frogs of the species *Leptodactylus latrans* (Table 2), one of which had been swallowed headfirst. Previous studies also indicate that the species feeds on frogs (Dixon et al. 1993, Marques 1998). At the Etá farm, individuals were found in January, May, June and December; one female had 38 primary follicles in May and another had ten secondary follicles in January. Previous observations indicate that it has a fecundity of 10–14 eggs and has a seasonal reproductive cycle (vitellogenic follicles in August–December and oviductal eggs in October–November; Costa et al. 2005). It may open its mouth, perform a frontal display by raising its head, forming an S-coil with the anterior part of its body, and inflating the gular region, strike, perform cloacal discharge and vibrate the tail (Marques et al. 2019, Martins et al. 2008; Table 3).

##### *Spilotes pullatus* (Linnaeus, 1758)

This is an aglyphous species of large size (maximum SVL = 1830 mm; *N* = 15). It was found in a pile of wood next to a house in an operating banana plantation (*N* = 4), in an abandoned banana plantation (*N* = 1), in peach palm plantations (*N* = 2), on the edge of the forest (*N* = 1), in a pasture (*N* = 1) and crossing a paved road in areas surrounded by forest and pasture (*N* = 2). Three run-over individuals were collected on an unpaved road near the edge of forest. The available information indicates that it is semi-arboreal and diurnal (Vanzolini et al. 1980, Marques and Sazima 2004, Bernarde and Abe 2006, this study). We found four rodent nestlings, all ingested headfirst, in the stomach of a specimen (Table 2). Previous studies with specimens from the Atlantic forest revealed that this snake feeds mainly on very small mammals and nestling birds (Marques et al. 2014). At Etá farm, one mating pair was found in September; two females had eight and 11 eggs in their oviducts, in September. Most adult individuals were observed in August and September (*N* = 7), while juveniles were observed in February (*N* = 3). Marques et al. (2014) reported oviposition for various localities in the Atlantic forest, restricted to the onset of the rainy season (October–November) and Bernarde and Abe (2006) observed juveniles in the same period in southwestern Amazon. Previous reports indicate that it lays 5–12 eggs (Amaral 1930, Hauzman and Costa 2005, Marques et al. 2014). The temperament of the individuals found varied greatly, from very docile to extremely aggressive. When handled, the individuals found opened the mouth (*N* = 1), vibrated the tail (*N* = 4), inflated the gular region (*N* = 10) and struck (*N* = 4) (Table 3). Previous studies indicate that it may also perform cloacal discharge and frontal displays by raising the head and forming an S-coil with the anterior part of its body (Martins et al. 2008, Marques et al. 2019).

#### Dipsadidae Bonaparte 1838

##### *Dipsas neuwiedi* (Ihering, 1911)

This is an aglyphous species of medium size (maximum SVL = 643 mm; *N* = 31). It was found mostly in the peach palm plantation (*N* = 15), with only one individual captured in a pitfall trap. It was found also around the houses (*N* = 5) and crossing the unpaved road at night (*N* = 5). One individual was found resting under the lid of one of the pitfall trap buckets during the day. We have no information on habitat use for the other five individuals. The available information indicates that it is nocturnal and semi-arboreal (Freitas 1999, Oliveira 2001, this study). Of the 31 specimens examined, ten had molluscs in their digestive tract (Table 2), most of them of the genus *Phyllocaulis* (*N* = 4), endemic to South America. In two cases the snakes had eaten Limax cf. flavus snails, an exotic European species, while in another, a *Meghimatium pictum* slug, an exotic Chinese species, both considered invasive. The available information indicates that it is a mollusc specialist (Freitas 1999, Oliveira 2001, Marques and Sazima 2004, this study). It was found throughout the year and one female had four oviductal eggs in March. The available information indicates that it has a seasonal reproductive cycle, with vitellogenesis occurring between July and December, and oviposition between August and February (Pizzatto et al. 2008). Barbo et al. (2011) reported a female with nine eggs. When handled, the individuals found at the Etá Farm performed cloacal discharge (*N* = 4), hid the head within the coiled body (*N* = 1), and triangulated the head (*N* = 19). Information available in the literature indicates that, besides being a supposed mimic of vipers such as *B. jararaca*, it may also raise the head and form an S-coil with the anterior part of the body (Martins et al. 2008) (Table 3).

##### *Echinanthera cephalostriata* Di-Bernardo, 1996

This is an aglyphous species of small size (maximum SVL = 399 mm; *N* = 3). Individuals were found crossing the road near forest and pasture areas during the day (*N* = 2), and moving in the leaf litter in the forest at night (*N* = 1). In a study at Serra do Medanha, Rio de Janeiro (Pontes et al. 2008), the species was found exclusively in forests. The available information indicates that it is diurnal, terrestrial, and cryptozoic (Martins et al. 2008, Barbo et. al. 2011, Hartmann et al. 2011, this study), and feeds on frogs (Marques et al. 2019, Marques et al. 2009, Forlani et al. 2010). At the Etá farm, it was found during the months of July, November and December. One female collected in the municipality of Iguape (50 km from the Etá Farm) had eight vitellogenic follicles in November (data provided by staff from the Butantan Institute). When handled, one of the individuals found raised the anterior part of the body (Table 3). It may also flatten its body and perform cloacal discharge (Martins et al. 2008, Marques et al. 2019).

##### *Erythrolamprus aesculapii* (Linnaeus, 1766)

This is an opistoglyphous species of medium size (maximum SVL = 827 mm; *N* = 5). Four individuals were found on an unpaved road: two moving near a pasture, one near an operating banana plantation and one crossing an unpaved road near the forest edge, all during the day. The last individual was found inside the forest. The available information indicates that it is terrestrial, cryptozoic and primarily diurnal (Bernarde and Abe 2006, França and Araujo 2006, Martins et al. 2008, this study). In the digestive tract of the examined specimens we found an individual of *D. neuwiedi*, swallowed headfirst in one specimen, and snake scales in another one (Table 2). It feeds primarily on other snakes, but juveniles also feed on very small lizards (Martins and Oliveira 1998, Bernarde and Abe 2006, França and Araujo 2006, this study). At the Etá Farm, a female had two vitellogenic follicles and two juveniles were found in June. The available information indicates that it has a fecundity of 3–8 eggs and a continuous reproductive cycle, with vitellogenesis occurring throughout the year (Marques 1996a, Marques and Sazima 2004). This snake is a supposed mimic of *M. corallinus*. Besides the similarity of the colour pattern, it flattens the body, hides the head, performs erratic movements, and raises the curled tail as in *M. corallinus*.

##### *Erythrolamprus miliaris* Linnaeus, 1758

This is an aglyphous species of medium size (maximum SVL = 1000 mm; *N* = 74). It was found in all sampled vegetation types and captured, in most cases, in pitfall traps (17 in the abandoned banana plantation, 22 in forest and 23 in the peach palm plantation); it was also found in other disturbed areas (*N* = 13). When found in the traps, all individuals submerged into the water accumulated in the buckets. Most individuals were seen in the abandoned banana plantation, moving or resting in the undergrowth (*N* = 4), or foraging at the edge of flooded vegetation (*N* = 1); in the forest, moving on the ground or on the leaf litter (*N* = 2); and, in the peach palm plantation, in water puddles (*N* = 2) or undergrowth (*N* = 1). In visual searches and occasional encounters, individuals were also found at the edge of the forest (*N* = 1), in pasture areas (*N* = 3), in a swamp (*N* = 1), and around houses (*N* = 2). Some individuals were captured on the unpaved road, always near flooded areas (*N* = 3) and one adult individual was found in a puddle in an open area near the forest edge. Two juveniles were captured in very disturbed habitats, one in the sink in a house at 12:00 h and another in an operating banana plantation, moving over rocks at 15:30 h. Additionally, four individuals were found on the unpaved road, one crossing it near a swamp during the day, and another run over by a car near the peach palm plantation at 06:00 h. Observations of individuals moving were always in daytime. The available information indicates that it is semi-aquatic and both diurnal and nocturnal (Sazima and Manzini 1995, Yanosky et al. 1996, Martins et al. 2008, Torello and Marques 2017, this study). More than 90% (*N* = 38) of specimens whose digestive tract contents were analysed contained frogs (Table 2). One snake captured in a small homemade water tank contained a specimen of *Synbranchus marmoratus* in its digestive tract, swallowed by the tail, while another snake, captured in a pitfall trap, had a specimen of *Placosoma glabellum*, also ingested by the tail (Table 2). The available information indicates that it feeds mainly on amphibians, but also on fish, tadpoles, amphisbaenians and lizards (Achaval and Olmos 1997, Carreira 2002, Marques and Sazima 2004, Toledo et al. 2007, this study). At the Etá Farm it was found throughout the year, with a higher incidence in hot and rainy months. Two females showed 5–12 vitellogenic follicles from April to October, and one female had 5 oviductal eggs in October. Juveniles (< 400 mm; *N* = 36) were mainly observed during the rainy season. Pizzatto and Marques (2006) reported different types of reproductive cycle for this species in different regions of the Atlantic Forest: a continuous reproductive cycle on the coast at the northern parts of its distribution (southern Bahia state), and a seasonal one in the southern regions, both in the interior and in coastal regions (São Paulo and Paraná states), with vitellogenesis and oviposition from September to February, and births at the end of the rainy season. It has a fecundity of 5–17 eggs and reaches sexual maturity at 12 months of age, at the earliest (Vitt 1992, Achaval and Olmos 1997, Pizzatto and Marques 2006, this study). When handled, the individuals found at the Etá Farm flattened (*N* = 2) or rotated (*N* = 5) the body, raised the head while flattening the body (*N* = 2; see Menezes et al. 2015), performed cloacal discharge (*N* = 20), and triangulated the head (*N* = 3), as previously reported in the literature (Martins et al. 2008, Marques et al. 2019) (Table 3).

##### *Helicops carinicaudus* (Wied & Neuwied, 1825)

This is an aglyphous species of medium size (maximum SVL = 623 mm; *N* = 12). Thirteen individuals were found in the field; of those, three in the peach palm plantation, with one individual captured in a pitfall trap, and the other three in a stream at the edge of forest, in the water, all apparently active in late afternoon. Three individuals were captured on the unpaved road, all moving during the day. The other captured individuals (*N* = 7) moved across floodplains on the edge of the forest, also in late afternoon. Literature records of activity were made during both day and night (Marques and Sazima 2004, Hartmann et al. 2009b). Of the specimens examined, three had stomach contents: two of them had frogs and the other had a fish (*Characidium* sp.; Table 2). The available information indicates that the diet of this species consists mainly of fish, although it may also capture frogs (mainly leptodactylids; Albolea 1998, Marques and Sazima 2004, this study). Individuals of the species were found mainly between August and November. One female had one vitellogenic follicle in August, while two others had 11 and 13 in October and November, respectively. Two juveniles (< 350 mm) were found in March and August. The available information indicates that it has a seasonal reproduction, with vitellogenesis occurring from September to December, embryos from November to March, and juvenile recruitment between February April in Atlantic forest regions (Marques 1998, Nogueira and Marques 1998, this study). Fecundity varies between 7 to 26 embryos (Nogueira and Marques 1998). When handled, individuals found at the Etá Farm struck (4) and bit (1). Previous observations indicate that it may also flatten the body, hide the head, and perform cloacal discharge (Marques et al. 2019) (Table 3).

##### *Oxyrhopus clathratus* Duméril, Bibron & Duméril, 1854

This is an opisthoglyphous species of medium size (maximum SVL = 710 mm; *N* = 12). It was found mainly around houses trying to climb walls at dusk (*N* = 6), run over on the unpaved road next to the forest edge (*N* = 2), and moving on the ground in the forest at night (*N* = 1). One adult male was caught while crossing the unpaved road near the edge of forest at 05:30 h. Three individuals were captured in other disturbed habitats. The available information indicates that it is terrestrial and both diurnal and nocturnal (Hartmann and Giasson 2008, Martins et al. 2008, Barbo et al. 2011; this study). One individual regurgitated a marsupial (*Monodelphis americana*) (Table 2), inside one of the pitfall traps. The available information indicates that it feeds mainly on mammals, but juveniles feed primarily on lizards (Morato 2005, Hartmann et al. 2009b, Alencar 2010, Gaiarsa et al. 2013, this study). Most individuals found at the Etá Farm were juveniles (< 500 mm), found between June and September. One female collected in the municipality of Cananéia (78 km from the Etá Farm) had 17 vitellogenic follicles in April (data provided by the staff of Butantan Institute). The available information indicates that reproduction is seasonal, with the reproductive peak occurring at the onset of the rainy season in the Atlantic forest (Marques and SaziMa 2004); fecundity ranges from four to 16 eggs (Gaiarsa et al. 2013). When handled, one of the individuals found thrashed the body. This species is a supposed imperfect mimic of *Micrurus* spp. and the defensive behaviour also includes hiding the head and making erratic movements (Martins et al. 2008, Marques et al. 2019) (Table 3).

##### *Sordellina punctata* (Peters, 1880)

This is an aglyphous species of small size (maximum SVL = 461 mm; *N* = 7). It was captured in a pitfall trap in the forest (*N* = 1), crossing an unpaved road near the margins of the Etá River (*N* = 4), and in a pasture area (*N* = 1), at dusk (*N* = 3) and at night (*N* = 1). One individual was captured in another disturbed habitat. One individual had been ingested by an individual of *B. jararacussu* at the edge of the forest, at night. The available information indicates that this is a semi-aquatic, primarily diurnal species (Marques et al. 2019, Pereira et al. 2007, Marques et al. 2009, this study). A leech was found in the digestive tract of one specimen from the Etá Farm (Table 2). The available information indicates that it feeds primarily on Oligochaetes (earthworms and leeches) and eventually on caecilians (Proctor 1923, Marques 1996c, Marques et al. 2009, this study). At the Etá Farm, it was found active mainly in the hotter and rainier months, except for one individual moving across the unpaved road in June. Data obtained from preserved snakes indicate that it has a seasonal reproductive cycle (Marques, 2001). When handled, two of the individuals found performed cloacal discharge. Information available in the literature indicates that it may also flatten the body (Marques et al. 2019).

##### *Taeniophallus bilineatus* (Fischer, 1885)

This is an aglyphous species of small size (maximum SVL = 258 mm; *N* = 2). Two individuals were found, one captured in a pitfall trap in the forest, the other moving along the forest edge in the morning. The available information indicates that it is terrestrial and diurnal (Marques et al. 2019, Hartmann et al. 2009b, Forlani et al. 2010, this study). The second individual found was in a forest in July. It feeds on frogs and lizards (Di-Bernardo and Lema 1990, Marques and Sazima 2004). Apparently, it has a seasonal reproductive cycle (Marques and Sazima 2004). It may flatten the body and perform cloacal discharge (Marques et al. 2019) (Table 3).

##### *Tomodon dorsatus* Duméril, Bibron & Duméril, 1854

This is an opisthoglyphous species of small–medium size (maximum SVL = 540 mm, *N* = 3). One adult female was found in July around a house, one adult male was found on the unpaved road in December and another adult male was found run-over near the peach palm plantation in January. The available information indicates that it is terrestrial, cryptozoic and diurnal (Marques and Sazima 2004, Martins et al. 2008, Araujo et al. 2010). It feeds on molluscs (Marques et al. 2019) and some authors have suggested that it may show aggregation, perhaps related to food availability (molluscs; Bizerra 1998, Franco et al. 2006). One female collected in the municipality of Itariri (82 km from Etá Farm) had 13 vitellogenic follicles in November (data provided by the staff of Butantan Institute). The available information indicates that it has a seasonal reproductive cycle, with vitellogenesis occurring mainly by the onset of the rainy season, embryos throughout the rainy season and litter size ranging from 4 to 26 (Bizerra et al. 2005, Barbo et al. 2011). It may flatten the body, strike, raise the head and form an S-coil with the anterior part of the body, hide its head and display the buccal mucosa (Martins et al. 2008, Marques et al. 2019) (Table 3).

##### *Xenodon neuwiedii* Günther, 1863

This is an aglyphous species of medium size (maximum SVL = 555 mm; *N* = 6). It was captured in a pitfall trap in the forest (*N* = 1). One juvenile was captured as it moved through the leaf litter during the day, at 08:20 h, and one adult male was captured while crossing an unpaved road at 10:50 h. One individual was found on the peach palm plantation and another on an unpaved road, near a pasture area, during the day. The last individual was caught in unpaved road close to disturbed areas. The available information indicates that it is diurnal and terrestrial (Hartmann et al. 2009b, Forlani et al. 2010, this study). Of the specimens that had their digestive tracts examined, one had the remains of frogs: one *Rhinella icterica* and the legs of a *R. hoogmoedi* (Table 2). The available information indicates that it feeds mainly on frogs (mainly *Rhinella* spp.), lizards being an occasional prey (Silva and Rodrigues 2001, Marques and Sazima 2004, Hartmann et al. 2009b, Costa et al. 2012, this study). Most individuals found at the Etá Farm were juveniles (except for one adult male) and were found between November and December, except for one juvenile found in May. The available information indicates that it reproduces throughout the year (Jordão 1996, Condez et al. 2009) and its fecundity can reach 14 eggs (Hartmann et al. 2009a). When handled, individuals found at the Etá Farm flattened the body (*N* = 1) or struck (*N* = 1). The available information indicates that, besides being a supposed mimic of vipers such as *B. jararaca*, it may also triangulate the head, raise the head and form an S-coil with the anterior part of the body (Martins et al. 2008), or elevate the head while compressing the body (Greene 1979) in a manner similar to Old World elapids (Table 3).

#### Elapidae Boie, 1827

##### *Micrurus corallinus* (Merrem, 1820)

This is a proteroglyphous species of medium size (only juveniles were captured, with maximum SVL = 251 mm; *N* = 5; adults exceed 900 mm in total length; Roze 1996). One individual was captured in a pitfall trap in the peach palm plantation, another was found inside a house, and a third was caught crossing an unpaved road near the forest edge in the early morning. The other three were active during the day in unpaved roads close to disturbed habitats. It is primarily diurnal, and it forages on the ground or in underground galleries capturing caecilians, amphisbaenians, lizards and other snakes (Roze 1996, Marques and Sazima 1997, Banci et al., 2017). Only juveniles were found at the Etá Farm (< 400 mm), in April, July and August. The species has a seasonal reproductive cycle with mating and vitellogenesis occurring at the beginning of the rainy season (Marques 1996b, Almeida-Santos et al. 2006, Marques et al. 2006). Fecundity ranges from 2–12 eggs (Marques 1996b). When handled, one individual from the Etá Farm raised the curled the tail. This coral snake flattens its body, hides its head, performs erratic movements and elevates its head, while compressing its body, and raises the curled tail (Greene 1979, Marques et al. 2019) (Table 3).

#### Viperidae Laurenti, 1768

##### *Bothrops jararaca* (Wied, 1824)

This is a solenoglyphous species of large size (maximum SVL = 1220 mm; *N* = 23). It was found in all sampled vegetation types (eight individuals in peach palm plantation, four in forests, and one in the abandoned banana plantation), but never in pitfall traps. It was also found in operating banana plantations (*N* = 5). The other five individuals were caught in other disturbed habitats. Most individuals found were coiled up in the undergrowth during the day, in the peach palm plantation; one individual was found around houses and two on an unpaved road, one of them near the forest edge and the other in a pasture area. One adult male was found moving on the ground in the afternoon (15:00 h) in the abandoned banana plantation, one juvenile was found moving over a bromeliad on a fallen trunk at night (see also Marques 1998) and one adult male was found moving on the forest ground at 22:00 h. The available information indicates that it is semi-arboreal and primarily nocturnal (Sazima and Manzani 1995, Alves et al. 2000, Martins et al. 2001, Forlani et al. 2010, this study). In the digestive tracts of three specimens examined we found rodents, one of them belonging to the tribe Akodontini (Table 2). The available information indicates that it feeds mainly on rodents and amphibians, in addition to lizards, birds and centipedes, with a relatively higher consumption of ectothermic prey by juveniles and endothermic prey by adults (Sazima and Manzani 1995, Martins et al. 2002, Hartmann et al. 2009b); there have been reports of necrophagy in this species (Sazima and Strüssmann 1990). Five females from the Etá Farm had 13–35 vitellogenic follicles, throughout the year. One gravid female captured in January contained 10 fully formed embryos (SVL = 191 ± 15.23 mm, weight = 7.48 ± 1.29 g). Juveniles (< 400 mm) were mainly observed in the hotter and rainier months of the year (October to February). The available information indicates that it has a biennial, seasonal reproductive cycle, with vitellogenesis occurring between autumn and winter, ovulation probably in early spring, copulation at the beginning of the dry season and pregnant females from November to March (Janeiro-Cinquini 2004, Almeida-Santos and Salomão 2002, this study). Fecundity varies from 3–36 offspring and gestation may last 152–239 days (Alves et al. 2000, Janeiro-Cinquini 2004, Sazima 1992, Almeida-Santos and Salomão 2002, Costa et al. 2010, this study). It was found throughout the year at the Etá Farm. When handled, individuals from the Etá Farm vibrated the tail (*N* = 6) and struck (*N* = 4). The Information available in the literature indicates that it may also flatten its body and perform cloacal discharge (Marques et al. 2019) (Table 3).

##### *Bothrops jararacussu* Lacerda, 1884

This is a solenoglyphous species of large size (maximum SVL = 1150 mm; *N* = 47). It was found in all sampled vegetation types (23 individuals in the peach palm plantation, six in forest and four in banana plantations), coiled up in the undergrowth, often at the base of the peach palms (*N* = 17), in open areas or on the leaf litter in the forest (*N* = 4); only two juveniles were captured in pitfall traps, in the abandoned banana plantation and the peach palm plantation. Nine individuals were found on the unpaved road, three of them at the edge of forest, one of which had just ingested an individual of *Sordellina punctata*, and three near pasture areas. The other five individuals were caught in other disturbed habitats. The available information indicates that it is terrestrial and frequently found close to watercourses; it has mostly nocturnal activity, although juveniles and eventually adults may hunt during the day (Martins et al. 2002, Marques and Araujo 2011, M. Martins, personal observation). Approximately 63% of the specimens (*N* = 12) whose digestive tracts were examined had rodents (Table 2) that, in all cases where it was possible to assess, had been ingested headfirst. One specimen contained the bones of an opossum. The population of the Etá Farm seems to show an ontogenetic change in diet, with juveniles feeding mainly on ectothermic animals, including a snake (*Sordellina punctata*), frogs of the families Hylidae and Leptodactylidae, and a centipede, and adults feeding primarily on small mammals. However, adult individuals may occasionally consume ectothermic prey, as in the case of one adult male containing a rodent and a leptodactylid frog in its digestive tract (Table 1). The available information indicates that it feeds on centipedes, frogs, lizards and mammals, with ontogenetic variation in diet, with juveniles mainly feeding on ectothermic animals (especially frogs) and adults mainly feeding on small mammals (Lema et. al. 1983, Martins et. al. 2002, Hartmann et al. 2009b, this study). At the Etá Farm it was found throughout the year; however, most individuals were found in the hotter and rainier months (October–April). Two females had vitellogenic follicles in June and July and one had secondary follicles in January. Most juveniles were observed in December and February. The available information indicates that the reproductive cycle of this species is seasonal; mating occurs in May and June, and births are concentrated in March, with 13–37 hatchlings (Marques 1998, Almeida-Santos and Salomão 2002). When handled, individuals from the Etá Farm struck (*N* = 2) and vibrated the tail on the ground (*N* = 5). Information available in the literature indicates that it may also flatten its body and perform cloacal discharge (Marques et al. 2019) (Table 3).

## Discussion

The snake assemblage of the Etá Farm region has a species composition similar to those of other studied assemblages in the Ribeira River Valley (e.g., Marques 1998, Forlani et al. 2010). The new records for Sete Barras Municipality (*C. fuscus*, *C. laevicollis*, *E. cephalostriata*, *Spilotes pullatus*, and *T. bilineatus*) were already expected to occur in the region, because they are typical Atlantic Forest species and their distributions overlap Sete Barras region (Nogueira et al. 2019). All species that potentially occur in the Etá Farm region are typical forest species (Marques et al., 2019, Nogueira et al. 2019) and most of them are semi-arboreal (Marques and Cavalheiro 1998, Martins et al. 2008, Antunes and Haddad 2009, Hartmann et al. 2009ab, Marques et al. 2019) and though most species found in the field present diurnal activity, when species of potential occurrence are added, most species of the assemblage are nocturnal (Martins et al. 2008, Antunes and Haddad 2009, Hartmann et al. 2009ab, Marques et al. 2019). The main prey of these species are amphibians (*C. bicarinatus*, *C. foveatus*, *E. cyanopleura*, *E. undulata*, *T. persimilis*, *T. nattereri*, and *T. paucisquamis*; Dixon et al. 1993, Antunes and Haddad 2009, Marques et al. 2019), slugs (*D. albifrons*, *D. alternans*, *D. indica* and *D. variegata*; Sazima 1989, Forlani et al. 2010, Marques et al. 2019), mammals and birds (*C. cropanii*, *C. hortulanus*; Marques and Cavalheiro 1998, Marques et al. 2019), elongate vertebrates (*C. plumbea*; Gaiarsa et al. 2013), and amphibians and lizards in *I. cenchoa*, *S. pulcher*, and *T. serra* (Marques et al. 2019). As for the conservation status of snakes from the Etá Farm region, *Corallus cropanii* is categorised as Endangered (EN) in the red list of the International Union for the Conservation of Nature (IUCN 2019), in the Brazilian red list (ICMBio 2018), as well as in the São Paulo State red list (São Paulo 2019). It is known from only five localities and the most recent published record is from Sete Barras (Machado-Filho et al 2011).

Of the species recorded in the study area, most used both forested (or were at least observed in forest edges) and open areas, except for *C. exoletus*, *D. neuwiedi*, *S. pullatus*, and *T. dorsatus*, which were observed only in disturbed areas (banana and peach palm plantations). The peach palm plantations, surrounded by forested areas may be functioning as routes from one edge to the other of the forested areas, and as foraging sites (as they have large quantities of frogs and molluscs; see Fiorillo et al. 2018). However, the edges of a given habitat tend to be hostile to organisms adapted to living in its interior and may contain both competitors and predators (Andrén and Angelstam 1988, Chalfoun et al. 2002). Even so, some snakes from the region of the Etá Farm seem to benefit from using these areas.

The colubrids of the assemblage, as well as the xenodontines, are mostly anuran specialists (Marques et al. 1998, Marques et al. 2019, this study), except for *S. pullatus*, which differed from other colubrids by showing a diet based mainly on small mammals and nestling birds (Marques 1998, Marques et al. 2019, this study). The anuran prey and substrate use differ among the diurnal frog-eating species. *Chironius* spp. prey on leptodactylid and hylid frogs at various substrate heights, *X. neuwiedii* search mainly by *Rhinella* spp. in the forest ground and in disturbed areas and *E. cephalostriata* and *T. bilineatus* consume mainly small frogs commonly found amid the leaf litter, such as Adenomera cf. marmorata, *Haddadus binotatus*, *Ischnochnema* sp., and *Physalaemus spiniger* (Fiorillo et al. 2018), and occasionally their eggs (e.g., *E. cephalostriata*, see Moura-Leite et al. 2003).

The nocturnal and terrestrial species, *B. jararaca*, *B. jararacussu*, and *O. clathratus* show similar diet and were all found in both open, disturbed areas and in forested areas. Although marsupials (e.g., *Monodelphis americana*) were restricted to forested areas, rodents were abundant in both habitat types. However, juveniles of these species feed on ectothermic prey (*O. clathratus* feeds on lizards and *Bothrops* spp. feeds mainly on frogs). It is known that *O. clathratus* is occasionally found in open and disturbed areas (Di-Bernardo et al. 2007, Hartmann and Giasson 2008, Hartmann 2009b). Being an almost strictly terrestrial species (Hartmann and Giasson 2008, Hartmann et al. 2009a, Barbo et al. 2011), probably it is not as restricted to forested habitats as other species that use arboreal substrates more often (e.g., *Chironius* spp.). However, it is important to consider that even the disturbed habitats in which the species was found, were surrounded by forest. In addition, the disturbed habitats sampled (banana and peach palm plantations) presented a considerable abundance of frogs (see Fiorillo et al. 2018), which in turn would favour the foraging of *Bothrops* species. The malacophagous species, *D. neuwiedii* and *T. dorsatus*, show terrestrial behaviour, but distinct daily activities (nocturnal for the former, diurnal for the latter), and both are found in open areas where the molluscs are abundant (personal observation). Two of the slug species consumed by *D. neuwiedii* are invasive species, the European Limax cf. flavus and the Chinese *Meghimatium pictum*. Thus, these malacophagous snakes can potentially control the populations of invasive molluscs.

Although annelids have been previously reported for the diet of *S. punctata* (Marques 1998, Marques et al. 2019), we here provided the first report of consumption of leeches by this species. This prey, earthworms (including aquatic giant earthworm) and one caecilian (previously reported by Proctor, 1923) confirm that this snake forages in aquatic habitats.

Most individuals were found during the hot and rainy season from September to March, when most species show reproductive activity, as may be seen by the presence of vitellogenic follicles for some species and mating (e.g., *S. pullatus*). This seasonal activity peak has been reported for other assemblages of Neotropical snakes (Strüssmann and Sazima 1993, Martins and Oliveira 1998, Marques 1998, Sawaya et al. 2008b, Pontes et al. 2009) and seems to reflect the effect of environmental variables favouring snake metabolism for the development of eggs or offspring. The exceptions were *E. aesculapii*, which had vitellogenic follicles in July; *E. miliaris*, which also presented secondary vitellogenic follicles from April to August, although smaller in the hottest period of the year; and the vipers *B. jararaca* and *B. jararacussu*, which showed vitellogenic follicles in both the hot and rainy season and during the month of June. Females of *E. aesculapii* and *X. neuwiedii* show vitellogenic follicles in every month of the year (Jordão 1996, Marques 1996a, Pizzatto et al. 2008). These two species belong to the tribe Xenodontini and continuous reproductive cycles may be conservative in this lineage of snakes (Pizzatto et al. 2008). Additionally, the type of resource used by these species may enable a continuous reproductive cycle (Vitt and Vangilder 1983, Seigel and Ford 1987), as these species feed on prey that are abundant throughout the year (Seigel and Ford 1987, Roberto et al. 2011).

The reproductive cycle of another member of the tribe Xenodontini, *E. miliaris*, varies along its distribution and, although vitellogenic follicles were observed from April to August (two individuals), the population of the Etá Farm region is characteristic of the southern coastal region of the Atlantic Forest, where the reproductive cycle of this species may be seasonal (Pizzatto and Marques 2006). This reproductive peak during the hotter and rainier months, as well as with other species in the assemblage (members of the genus *Chironius*), may be related to the temporal distribution of frogs, because, as in other species of the genus (e.g., *E. poecilogyrus*, see Alencar and Nascimento 2014), female *E. miliaris* apparently do not stop feeding during vitellogenesis until the deposition of the eggs (two females containing secondary follicles and one female with eggs in the oviducts had stomach contents), a characteristic that may be related to the possibility of foraging during the reproductive period (Winne et al. 2006, Dyke et al. 2012). Alternatively, Oliveira and Martins (2001) suggested that snakes, as well as frogs, may simply be responding to the same environmental variables (or set of variables) and, thus, their activity peaks coincide in time.

The results described herein for the reproduction of *B. jararaca* and *B. jararacussu* corroborate the results of other studies that describe the reproductive phenology for the genus *Bothrops* (Almeida-Santos and Salomão 2002, Janeiro-Cequini 2004). These studies suggest that copulation occurs from April to September, when the animals are in vitellogenesis, there is a reduction of ovarian mass from October to March, probably due to ovulation and advanced pregnancy occurs during the rainy season (see the account of *B. jararaca* in Results). These patterns occur due to the storage of sperm in females and late fertilisation, which allows mating to occur in one season and follicular development, fertilisation and parturition to occur in another. Moreover, it provides females with the possibility of repeated fertilisation in a single mating event (Birkhead and Moller 1993, Marques 1996b).

Most defensive tactics observed at the Etá Farm were apparently aimed at visually oriented predators such as birds (especially birds of prey), important predators of Neotropical snakes, and some mammals (Sazima 1992, Martins and Oliveira 1998, Martins 1996, Martins et al. 2008). However, in the case of mammals, these tend to show nocturnal activity and, thus, to use mainly their sense of smell and hearing as the main ways to locate their prey (Martins 1996). Thus, a defensive tactic such as cloacal discharge (employed by 70% of the species in the region, considering field observations and literature data; see Table 3) could be more effective. Alternatively, Martins (1996) suggest that defensive tactics seem to correlate with phylogeny, therefore, cloacal discharge (a defensive behaviour with apparently low energy costs) may have been the most frequent behaviour observed simply because it is a common, well established, widespread behaviour among snakes in their evolutionary history.

Another defence shown by many species was cryptic colouration (82%), which is common for diurnal species (58% of the assemblage; Martins & Oliveira, 1998). In contrast, only one species showed aposematic colouration (*M. corallinus*) and two (*E. aesculapii* and *O. clathratus*) are supposed mimics of the coral snakes (Greene and McDiarmid 2005; Martins et al., 2008). Many of the trends in defensive tactics observed in this study are similar to those found by previous works in the Central Amazon (Martins 1996, Martins and Oliveira 1998, Martins et al. 2008), including: (1) inaccessibility (e.g., see the account of *C. laevicollis*); (2) many species employ tactics like fleeing, compressing their bodies, and biting; (3) all diurnal species are cryptic, except for the aposematic species, *M. corallinus*, and the supposed coral snake mimic, *E. aesculapii* (additionally, although not observed in this study, there are reports in the literature of the same defensive behaviours of *M. corallinus* performed by *E. aesculapii*); and (4) head triangulation was commonly used by supposed mimics of pitvipers (e.g., *D. neuwiedii*, *X. neuwiedii*; Greene and McDiarmid 2005), but also by *E. miliaris*.

A poorly documented visual defensive behaviour was reported for *E. miliaris* at Fazenda Etá (Menezes et al. 2015; this study). Two individuals (one juvenile and one adult) were observed simultaneously raising and compressing the anterior part of their body, a behaviour commonly performed by Old World elapids and previously reported for other Neotropical snakes, for example, species of the genera *Thamnodynastes*, *Hydrodynastes*, and *Xenodon* (Franco et al. 2003, Young and Kardong 2010, Kahn 2011). It is likely that this behaviour has the same goal of intimidating the predator as other frontal displays previously described, such as raising the head and inflating the glottis, which alter the predator’s perception of the size of the individual that performs them (Greene 1988, 1997, Young and Kardong 2010).

## Conclusions

The region of the Etá Farm harbours a rich snake fauna that is similar in composition to those of other snake assemblages in the Ribeira River Valley and includes one threatened species. This study contributed to the knowledge of the snake fauna of this region also by providing five new records for the Sete Barras Municipality. The detailed natural history information provided herein may be used in the assessment of the conservation status of these snakes and in the definition of action plans aiming to conserve this rich and biologically diverse fauna.

### Keys to families and species of snakes from Sete Barras region, southeastern Brazil

**Table:** 

1	Loreal pit present; solenoglyphous dentition; keeled dorsal scales	** Viperidae **
–	Labial pits present; aglyphous dentition; smooth dorsal scales; large size	** Boidae **
2	Labial pits absent; small size; stout body; usually 21 or 23, rarely 25 midbody dorsal scale rows; 164–183 ventral plates; 15–19 maxillary teeth	**Tropidophiidae (*Tropidophis paucisquamis*)**
–	Proteglyphous dentition; small black eyes; loreal shield absent; coral colour pattern, with single black rings between two narrow white rings	**Elapidae (*Micrurus corallinus*)**
3	Aglyphous or opistoglyphous dentition; top of head covered by large, distinct and symmetrical scales	**Colubridae and Dipsadidae**

**Viperidae**

**Table:** 

1	Dorsal spots in inverted “V” shape, bordered by lighter colours; belly lighter with irregular spots; 20–37 dorsal scale rows; 170–216 ventral plates; 44–71 subcaudal plates; 6–10 supralabial scales	***Bothrops jararaca***
–	Trapezoid dorsal spots, bordered by lighter colours; light-yellow belly; 23–29 dorsal scale rows; 166–186 ventral scales; eight supralabial scales	***Bothrops jararacussu***

**Boidae**

**Table:** 

1	Olive-beige dorsum, with dark-brown rhomboidal spots from the neck to half of the tail; yellow ventral scales; 29–32 dorsal scale rows at midbody	***Corallus cropanii***
–	Extremely variable dorsal patterns, from grey to brown, yellow to orange and red; cream to light grey belly; 47–63 dorsal scale rows at midbody	***Corallus hortulanus***


**Colubridae and Dipsadidae**

**Table:** 

1	Even number of dorsal scale rows	**2**
–	Odd number of dorsal scale rows	**7**
2	More than 14 dorsal scale rows at midbody; apical pits present; dorsal background black; yellow belly colour invades the dorsolateral region	***Spilotes pullatus***
–	10 to 12 dorsal scale rows at midbody; single cloacal plate	**3**
3	10 dorsal scale rows at midbody; dorsal colour brown with shades of olive; keeled paravertebral scales; maxillary teeth 39–51	***Chironius fuscus***
–	10 or 12 dorsal scale rows at midbody; apical pit single and only on the neck scales	**4**
4	Adults with head, supralabial scales and anterior region of the body black; yellowish belly; juveniles are born completely green; 156–165 ventral scales; maxillary teeth 32–39	***Chironius laevicollis***
–	Divided cloacal shield	**5**
5	Eight posterior dorsal scale rows; anterior third of body olive green, turning to brownish in the other two thirds; light belly; 123–162 ventral scales; 111–160 subcaudal scales; 24–34 maxillary teeth	***Chironius exoletus***
–	Ten posterior dorsal scale rows.	**6**
6	Light green dorsum; belly light with shades of yellow; 163–174 ventral scales; 156–169 subcaudal scales; 32–37 maxillary teeth	***Chironius foveatus***
–	Dorsal colour olive green with a lighter vertebral stripe; yellow belly; 149–169 ventral scales; 121–157 subcaudal scales; 28–40 maxillary teeth	***Chironius bicarinatus***
7	17 or less dorsal scale rows at midbody	**8**
–	19 or more dorsal scale rows at midbody	**18**
8	15 dorsal scale rows at midbody	**9**
–	17 dorsal scale rows at midbody	**13**
9	Big black eyes; coral colour pattern; opistoglyphous dentition	***Erythrolamprus aesculapii***
–	Medium-sized eyes; colour pattern not coral-like	**10**
10	Top of head with several spots; aglyphous dentition; vertebral scale row distinctly larger than the other dorsal scales; belly with thin spots, forming irregular and rather interrupted longitudinal lines, 161–184 ventral scales; 56–83 subcaudal scales	***Dipsas neuwiedi***
–	Laterally compressed body; head extremely distinct from the body; large eyes	**11**
11	A pair of parallel spots on top of head; irregular dorsal spot pattern; slightly enlarged vertebral scale row; 11–15 maxillary teeth	***Dipsas albifrons***
–	A pair of white-bordered ocelli on top of head; beige dorsum, with dark round, well-defined blotches, thinly bordered by white	***Dipsas alternans***
12	Top of head blotched to mostly immaculate, but never with distinctive inverted U or V shaped blotches with light centres	***Dipsas variegata***
–	Head with or without large parietal spots, otherwise mostly immaculate; 16–26 dorsal blotches	***Dipsas indica***
13	Dark oral lining; opistoglyphous dentition; large fangs; 134–143 ventral scales; 31–28 subcaudal scales; eight or less maxillary teeth	***Tomodon dorsatus***
–	Light oral lining	**14**
14	Body uniformly black, with a series of light lateroventral spots; light spots on the supralabial scales; medium-sized eyes; subelliptical pupils; 135–174 ventral scales; 36–57 subcaudal scales	***Sordellina punctata***
–	Each dorsal scale with a lighter centre, with dark borders; medium-sized eyes; 142–171 ventral scales; 39–64 subcaudal scales	***Erythrolamprus miliaris orinus***
15	Well-defined line along the *canthus rostralis* from the snout to the post-ocular region; top of head, dark; yellow belly; 8–23 maxillary teeth	***Taeniophallus bilineatus***
–	Supralabial scales and chin region usually stained by black; no postocular stripe; 51–82 subcaudal scales; less than 140 ventral scales	***Taeniophallus persimilis***
16	Continuous lateral postocular stripe; white lateral line at the fourth dorsal row; light brown dorsum; yellow belly, with a pair of black dots on each ventral scale; 142–160 ventral scales; 80–100 subcaudal scales	***Echinanthera cephalostriata***
–	A pair of light spots on the occipital region; dark middorsal band on the neck, usually with irregular borders	***Echinanthera undulata***
17	Supracephalic dark colouration extends to the middle of the dorsum, creating a dark dorsal band that contrasts with the paravertebral ground colour at least on the neck; anterior part of the dark pleural band usually regularly edged	***Echinanthera cyanopleura***
–	Body strongly laterally compressed and long; head very distinct from the body; large eyes; elliptical pupil; vertebral dorsal scale row different from the paravertebral rows; dorsum brown with dark diamond-shaped blotches	***Imantodes cenchoa***
18	Single internasal shield, dark-brown or black dorsum; belly cream with two (sometimes three) medial rows of black semilunar marks; 130–148 ventral scales; 48–73 subcaudal scales	***Helicops carinicaudus***
–	Paired internasal scales	**19**
19	Head uniformly black; long snout; numerous bands along the body, uniformly distributed and not continuous on the belly; 183–221 ventral scales; 46–88 subcaudal scales	***Oxyrhopus clathratus***
–	Thin and laterally compressed body; head very distinct from the body; large red eyes; long, thin tail; anterior maxillary teeth longer than the rear ones; some vertebral scales are red or orange; the red spots on the dorsum occupy 4–7 scale rows	***Siphlophis pulcher***
20	Black or dark-brown colouration; juveniles have a white stripe on the head and a dark wine-red nuchal stripe; 198–243 ventral scales; 70–97 subcaudal scales; vertical pupils; smooth dorsal scales	***Clelia plumbea***
–	Non-globular eye; cylindrical body; thick neck; intensely pigmented gular region; 142–167 ventral scales.	***Thamnodynastes nattereri***
21	Dorsoventral compression of the body; oblique dorsal scale rows; aglyphous dentition; 6–14 maxillary teeth, with additional pair of large laminate rear fangs	***Xenodon neuwiedii***
–	Light brown dorsum, with square-shaped blotches; head, distinct from the body; laterally compressed body; slightly keeled dorsal scales	***Tropidodryas serra***

## Supplementary materials

Supplementary material 1 Specimen identifications Explanation note List of collected and/or examined specimens and their identifications and museums. https://binary.pensoft.net/file/405615This dataset is made available under the Open Database License (http://opendatacommons.org/licenses/odbl/1.0/). The Open Database License (ODbL) is a license agreement intended to allow users to freely share, modify, and use this Dataset while maintaining this same freedom for others, provided that the original source and author(s) are credited.Bruno F. Fiorillo, Bruno R. da Silva, Frederico Alcântara Menezes, Otavio A. V. Marques, Marcio Martins
